# Selection for florfenicol resistance at sub-MIC concentrations in *Pasteurella multocida*

**DOI:** 10.1371/journal.pone.0327115

**Published:** 2025-06-24

**Authors:** Abigail Hughes, Ludovic Pelligand, Dan I. Andersson, Andrew Mead

**Affiliations:** 1 Comparative Biomedical Sciences, Royal Veterinary College, London, United Kingdom; 2 Clinical Services and Sciences, Royal Veterinary College, London, United Kingdom; 3 Department of Medical Biochemistry and Microbiology, Uppsala University, Uppsala, Sweden; University of Veterinary and Animal Sciences, PAKISTAN

## Abstract

Antimicrobial resistance is a global threat to both human and animal health, and transfer of resistance between these spheres is recognised as a key concern for all species. Selection for resistance at sub-inhibitory antimicrobial concentrations has been characterised for some bacteria-antimicrobial combinations but there is little data from non-laboratory strains, and veterinary antimicrobials and bacterial species. Here, we demonstrate a minimum selective concentration of 0.06 mg/L (1/6 xMIC) for florfenicol in wild-type *Pasteurella multocida*, through competition experiments between a susceptible strain and a *floR*-resistant mutant. We also show that sub-inhibitory concentrations of florfenicol do not appear to significantly select for *de novo* resistance in *P. multocida* and present the challenges with adapting experimental protocols between bacterial species. These results have important implications for antimicrobial resistance selection at sub-inhibitory concentrations, method development for within-species differentiation in novel bacterial species, and application to policy regarding antimicrobial contamination in animal-feed.

## Introduction

Antimicrobial resistance (AMR) is a substantial and growing threat to both human and animal health. This issue must be tackled from a “One Health” perspective due to the recognised risk of transmission of the antimicrobial drug (AMD) resistant pathogens themselves as well as horizontal transfer of resistance genes (HGT) between species [1,2]. In addition, the resistance selection potential of low level (sub-inhibitory) concentrations of AMDs is increasingly being recognised as being relevant for emergence and maintenance of resistance in bacterial populations. Thus, pathogen transmission, HGT of resistance genes, and sub-MIC selection are important drivers of the AMR problem that need to be understood for implementation of efficient global control strategies [3,4].

Regarding sub-inhibitory selection, previous studies have shown that the minimum selective concentrations (MSC) for various AMDs in *E. coli* and *Salmonella typhimurium* range from 1/4 to 1/230 of the wild-type minimum inhibitory concentration (MIC). Moreover, fixation of *de novo* resistance mutations at AMD concentrations as low as 1/2000 xMIC can produce high-level AMR phenotypes [5]. Despite these insights, further research is needed to identify the risk of AMR selection in clinical strains of pathogens relevant to both human and animal health, such as *P. multocida.* This zoonotic pathogen is primarily associated with bovine and swine respiratory disease and fowl cholera, and is found in the respiratory tract of many species. Although the zoonotic transmission of *P. multocida* is most commonly through cat bites, contact through nasal secretions from infected cattle or swine may represent a human health risk [6]. Perhaps more important is the public health risk associated with HGT of mobile genetic elements harbouring AMR genes from *P. multocida* to more prevalent zoonotic bacterial species such as *E. coli* [7,8].

Florfenicol, a veterinary-specific broad-spectrum AMD, is widely used for treating respiratory disease in swine and cattle. As a fluorinated derivative of chloramphenicol, it inhibits protein synthesis by reversible binding to the 23S rRNA in the 50S subunit of the bacterial ribosome [9]. The florfenicol exporter gene *floR* is often plasmid-borne in *Pasteurellaceae,* alongside resistance genes to many other classes of critically and highly important AMDs. These include aminoglycosides and sulphonamides, classed as critical and highly important to human medicine by the WHO, and beta-lactam AMDs, one of the most commonly used classes across species [10–12]. Consequently, florfenicol selection pressure can drive co-selection for multiple resistance phenotypes, significantly impacting the use of AMD in human and veterinary medicine. HGT between bacterial species [7,8] further increases the risk of disseminating multiple AMR phenotypes in high-risk pathogens, such as *E. coli*.

Florfenicol is frequently administered to swine as medicated feed to manage respiratory infections caused by *P. multocida* and other pathogens. The European Food Safety Authority (EFSA) has highlighted the data gap surrounding the risk of low florfenicol concentrations that may be present in manufactured feed from contamination/carryover of a medicated batch [13]. This is part of a wider collaboration with the European Medicine’s authority to identify specific AMD concentration limits to prevent selection of AMR, recognising the critical importance of determining sub-inhibitory MSCs [14].

At the time of writing, the only MSC for florfenicol was determined in *E. coli* as 0.042 mg/L (or 1/100 of the ratio of MSC to the MIC of the susceptible strain; MSC/MIC_SUSC_) by comparison of growth rates at sub-MIC florfenicol concentrations from non-competitive isolated cultures of susceptible and resistant populations [15]. However, this approach is rather insensitive and does not account for inter-strain genetic differences which may affect the fitness cost [16]. Thus, to obtain conclusive MSC data, direct competition experiments between otherwise isogenic susceptible and resistant populations are required [14,17]. In the absence of further data regarding the MSC for florfenicol, EFSA have determined a highly conservative predicted MSC (PMSC) of 0.00125 mg/L by applying the ratio of 1/100 to the lowest MIC for *E. coli* from the available European Committee for Antimicrobial Susceptibility Testing (EUCAST) wild-type MIC distribution for florfenicol susceptibility [13].

In this study, the selection and enrichment of florfenicol-resistant mutants of a clinical strain of *P. multocida* was investigated. Competitive growth assays between the susceptible wild-type strain and the otherwise isogenic *floR-*resistant mutant were performed at five sub-inhibitory florfenicol concentrations, enabling determination of the MSC for enrichment of the resistant population. We also showed that selection for *de novo* florfenicol resistance in *P. multocida* is not easily achieved using serial passage methods developed for other bacterial species. This research has important implications for defining the risk of low florfenicol concentrations in feed residues, environmental contamination, or long-acting formulations. It also demonstrates the application of robust and simple techniques to assess the MSC in non-model bacteria.

## Results

### MIC results/Isolate susceptibility post transformation

Florfenicol MIC for the susceptible wild-type *P. multocida* strain (PM6) was 0.35 mg/L. Post-transformation (see section “Generation and characterisation of *floR* transformants”) a resistant isolate (PM6_R2) was defined by an increased MIC of 25.6 mg/L and the presence of the *floR* gene.

### No resistant isolates identified after 400 generations under sub-MIC selective pressure

#### Serial Passaging for *de novo* resistance.

To determine if *de novo* resistant mutants could be selected for from a susceptible population, 8 independent lineages of the susceptible wild type non-hypermutator *P. multocida* strain were serially passaged for 400 generations at 10 sub-MIC florfenicol concentrations (ranging from 1/4–1/2048 x MIC) and in the absence of florfenicol. Resistant isolates of >3x MIC of the susceptible strain were screened for by plating onto florfenicol-selective agar. No florfenicol resistant isolates were identified.

#### Spontaneous mutation frequency.

A standardised mutation rate for rifampicin of 4.5 x 10^−10^ per cell per division was calculated for the susceptible wild-type *P. multocida* strain, PM6. This mutation rate is 10-fold and 1000-fold lower than wild-type and hypermutator *E. coli* strains respectively [18]. Alongside inspection of cumulative mutant distribution plots to ensure validity of the data, PM6 was determined to not have a hypermutator phenotype.

To determine the likelihood of spontaneous florfenicol resistance mutation under no selection pressure, approximately 9 x 10^11^ CFU/mL of the susceptible wild-type strain were plated onto selective agar. A single resistant mutant was identified with an MIC of 1.4 mg/L, 4x the MIC of the ancestral strain.

### Enrichment of *floR-*resistant mutant at 1/6 xMIC (0.06 mg/L)

**Fig 1:**
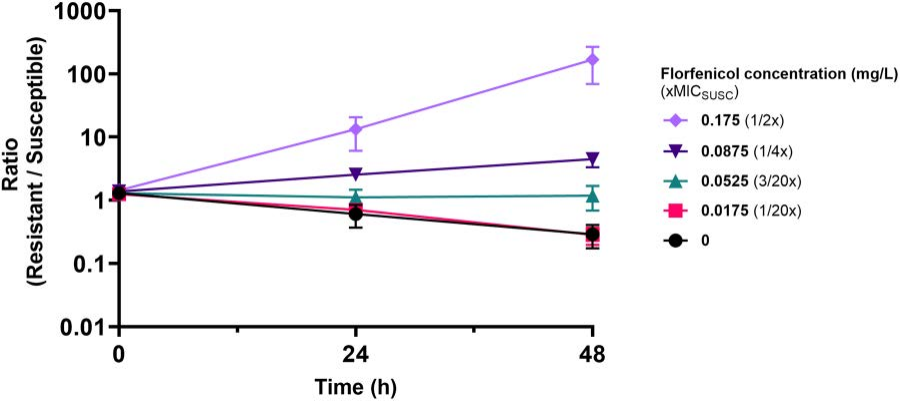
The ratio of resistant population/susceptible population over 48 hours. The ratio of the resistant population to the susceptible population for florfenicol concentrations 0 (black), 0.0175 (pink), 0.0525 (blue), 0.0875 (dark purple), and 0.175 (lilac) mg/L is depicted here as arithmetic mean and error calculated from 3 independent competition experiments. Concentrations are converted into factors of MIC_SUSC_ (0.35 mg/L). Passaging by 1000-fold dilution into fresh media at the same florfenicol concentration occurred at 24 hours, ratios were determined prior to passaging.

**Fig 2:**
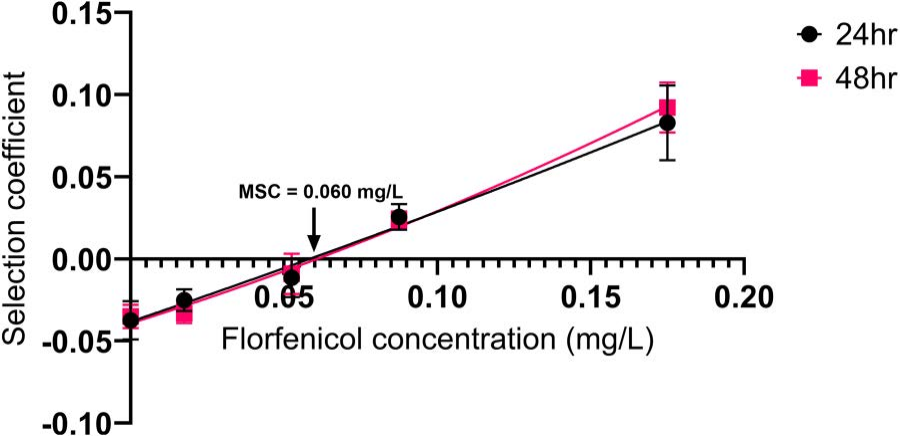
The mean selection coefficient versus florfenicol concentration. The mean selection coefficient calculated for florfenicol concentrations 0, 0.0175, 0.0525, 0.0875, and 0.175 mg/L (respectively 0, 1/20, 3/20, 1/4 and 1/2 xMIC_SUSC_) is depicted for 24 (black circles), and 48 (pink squares) hours. A second order polynomic nonlinear regression was fitted to each of the data sets: 24 hr (black line), 48 hr (pink line). Error bars represent the standard error. The MSC is the value where the selection coefficient is 0 (i.e., the concentration at which there is no difference in the susceptible: resistant population ratio over time). The mean MSC across 3 independent replicates was 0.06 mg/L which equals 1/6 xMIC_SUSC_.

To enable the detection of differences in fitness cost and account for competitive interaction, competition experiments were carried out between susceptible wild-type *P. multocida* (PM6) and a *floR*-plasmid carrying mutant (PM6_R2) of the same strain. The strains were isogenic except for the *floR*-carrying plasmid, isolated from a wild-type florfenicol-resistance *P. multocida* isolate (PM4) also containing genes encoding mobilisation proteins *mobA_2* and *mobC*, and replication proteins RepA, and RepC (S1 Fig), in the resistant strain. Strains were competed in batch cultures for 48 h, with 1000-fold serial passage at 24 h, at four sub-MIC florfenicol concentrations and in the absence of florfenicol. The ratio of resistant:susceptible strains at 24 h and 48 h, determined by enumeration of total and resistant populations from non-selective vs selective replica plate prints, is shown in Fig 1. The selection coefficients (sc), as slope of the change in ratio, are plotted against florfenicol concentration in Fig 2. The MSC, or concentration at which the fitness cost of the resistant strain is balanced by the florfenicol induced growth rate reduction on the susceptible strain, is defined as when the difference in growth between resistant and susceptible concentrations (i.e., the selection coefficient, *sc* equals 0). Interpolation of the nonlinear regression for y (*sc*) = 0 for each of 3 independent replicates demonstrated a mean MSC of 0.059 mg/L at 24hrs (Range: 0.037-0.074 mg/L) and 0.061 mg/L at 48 hours (Range: 0.046-0.078 mg/L), equivalent to 1/6 of the MIC of the susceptible strain.

## Discussion

This study is the first to report the MSC for any AMD in *P. multocida*. It expands on the florfenicol MSC determined for *E. coli* [15] by using competition experiments, contributing to resolving the knowledge gap for the MSC in this AMD of significant public and animal health interest to the EMA [13]. First, it is notable that we could not isolate any florfenicol resistant mutants *de novo*. The reason for this is at present unknown but may be related to the low mutation rate of *P. multocida* and/or a lack of feasible mutations that can confer resistance at ≥3 x MIC. By determining a specific MSC for florfenicol resistance selection in *P. multocida*, controls can be implemented in this widely used veterinary AMD to prevent not just florfenicol resistance selection critical for its continued efficacy in veterinary medicine, but also co-selection for resistance to AMDs critical to human medicine [10,11]. Maintaining continued efficacy of florfenicol in veterinary medicine is important for animal welfare and can prevent the use of AMD classes critical to human medicine. Tetracyclines and penicillins are alternative AMDs for the treatment of *Pasteurella multocida* [19,20], however they are the most frequently used AMDs in pigs and other food-producing animals [21,22] and humans [12,23], resistance to these classes remains high in important zoonotic pathogens [21]. Therefore, resistance developed to these AMD classes in animals may have greater public health implications from transfer to humans, as amphenicols are not commonly used systemically [24]. As the efflux pump, floR, is often encoded in transmissible plasmids alongside other resistance genes, florfenicol-mediated selection for bacteria containing these plasmids could lead to co-selection of resistance to critically important human AMDs.

**Fig 3:**
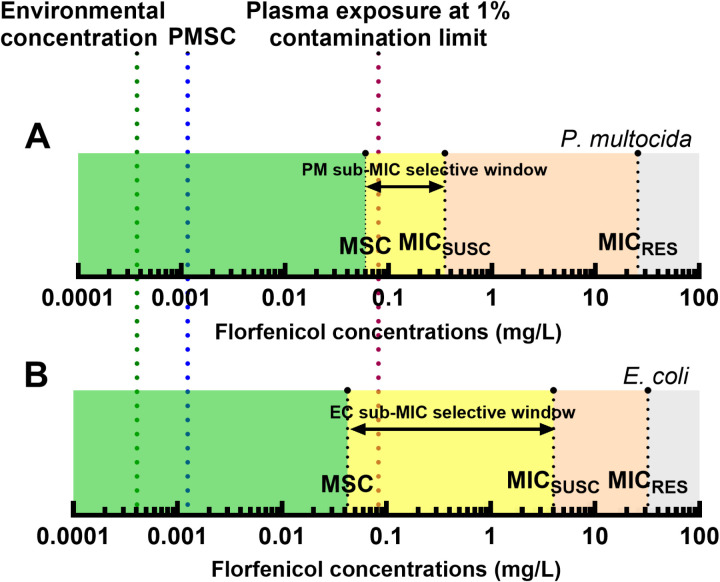
Resistance selection windows for floR-carrying P. multocida and cfr-carrying *E. Coli.* Florfenicol concentrations determining the traditional selective window (between MIC_SUSC_ and MIC_RES_, orange), sub-MIC selective window (yellow), and non-selective sub-MSC window (green) are depicted for *P. multocida* (A) and *E. coli* (B, from Zhang *et. al.* [15]). The florfenicol concentrations resulting in resistance selection are similar between both species although the majority is sub-MIC_SUSC_ for *E. coli* compared to supra-MIC_SUSC_ for *P. multocida*. The PMSC (blue dotted line, [13]) appears to be very conservative and is markedly different to the plasma/lung concentration predicted at the current maximum legally acceptable tolerated contamination limit for florfenicol in feed (red dotted line). Since the exposure is greater than the MSC reduction of the maximum residue limit is advisable. The florfenicol concentration in animal wastewater/effluent (green dotted line, [35]) is well below the MSC.

The mean MSC for *floR*-mediated florfenicol resistance in *P. multocida* was 0.06 mg/L. Zhang *et al.* [15] found a similar florfenicol MSC of 0.042 mg/L for *E. coli* with a *cfr*-carrying IncX4 plasmid (pSD11). However, the MIC_SUSC_ of florfenicol in *E. coli* (4 mg/L) is 10 times higher than *P. multocida* (0.35 mg/L) resulting in the significantly lower MSC/MIC_SUSC_ ratio: 1/100 x MIC_SUSC_ for *E. coli*-*cfr* compared to 1/6 x MIC_SUSC_ for *P. multocida-floR* (Fig 3). For tetracycline efflux pumps, the MSC has been determined as 0.015 mg/L (1/100 x MIC_SUSC_) and 0.025 mg/L (1/20 MIC_SUSC_) in *S. typhimurium* [25] and *E. coli* [26] respectively, demonstrating that here the absolute concentrations are also closer than the MSC/MIC_SUSC_ ratios. However, the K42R *rpsL* resistance mutation (allowing resistance to streptomycin) produced a MSC of 0.3 mg/L in clinical *E. coli* isolates [27] (MSC/MIC_SUSC_ ratio of 1/160) compared to 1 mg/L in *S. typhymurium* (MSC/MIC_SUSC_ ratio of 1/4) [25]. Therefore, the ratio of MSC/MIC_SUSC_ does not appear to be consistent even with the same resistance mechanism, which calls into question the validity of the current extrapolation of the MSC/MIC_SUSC_ ratio when predicting the MSC [14].

In the absence of experimentally derived MSCs specific to individual bacteria-AMD combinations, the EFSA uses the PMSC to calculate limits on feed residues. This is derived by extrapolating the MSC/MIC_SUSC_ ratio from one species to the minimum MIC from the distribution of the most susceptible species [14]. The PMSC value for florfenicol was determined as 0.00125 mg/L [13], which is 40-fold lower than the MSC determined here and therefore appears to be overly conservative (Fig 3). Further research into the species differences in MSC and the MSC/MIC_SUSC_ ratio could enable refinement of the prediction of MSC.

Comparison to MSCs determined with other AMDs is only possible by comparison of ratios due to different potencies of AMDs. The MSC/MIC_SUSC_ ratio determined here of 1/6 x MIC_SUSC_ falls within the range (1/4–1/230 x MIC_SUSC_) reported previously for enrichment of resistance to various AMDs in *E. coli* and *Salmonella* species [25,27,28]. By adding to this body of knowledge, we can develop an understanding of whether there is a typical MSC for an AMD class or resistance mechanism.

Zhang *et al.* [15] used independent (non-competitive) growth rates at sub-MIC florfenicol concentrations in both susceptible and resistant MG1655 *E. coli* isolates from OD600 measurement. The concentration at which the growth curves crossed was determined as the MSC. For *P. multocida*, independent growth curves determined by OD600, were indistinguishable for the susceptible (PM6) and resistant (PM6_R2) *P. multocida* isolates (S3 Fig). However, there was negative selection for the resistant isolate in the absence of florfenicol during co-culture. Measurement of viable bacterial counts enabled direct observation of this fitness cost, whereas the correlation of OD600 with bacterial population is limited and requires calibration for each bacterial species [29]. Co-culture also allowed the inclusion of competition dynamics between populations. This highlights the importance of using competitive growth curves and direct measurement of viable bacteria for estimation of fitness cost and MSC between strains when the fitness difference cannot be measured via optical methods, such as incompatibility of growth matrix [17].

Imazaki *et al.* [26] determined a tetracycline MSC in *E. coli* using population enumeration from “spot-plating” using selective agar. A trial of this method demonstrated that this would not be appropriate in *P. multocida* due to coalescence of the mucoid colonies, and the small number of colonies counted. Our study has demonstrated the utility of replica plate printing for differential bacterial enumeration when performing co-culture in wild-type bacterial species that are not routinely genetically manipulated. To understand the MSC and relationships between bacteria in co-culture generally, it is important to consider the feasibility of achieving fluorescent marking/genetic modification to differentiate strains via flow cytometric methods and physical characteristics such as morphology and growth rate. Replica plate printing may be used in a multitude of bacterial species and can be adapted to special growth conditions for fastidious organisms.

### Selective exposure in non-target environments

Defining the MSC is critical in assessing the risk of resistance selection in areas where low levels of florfenicol could expose non-target animals or environments containing *P. multocida*. Such exposure, could occur through feed cross-contamination, as highlighted by EFSA [13], or in pharmaceutical or animal effluent [17].

While the EU is in the process of identifying AMD-specific limits based on MSC data [14], the maximum permissible level for cross-contamination of an active antimicrobial in feed is currently 1% of the dose from the previous batch [30]. For a dosage concentration of 10 mg/kg per day [31], this would equate to a maximal legally acceptable non-target dose of 0.1 mg/kg per day. Allowing for a volume of distribution (at steady state) of 0.95 L/kg for florfenicol, 100% oral bioavailability [32] with 15% protein binding [33], this would lead to a daily exposure of an average free plasma concentration of 0.089 mg/L. Although lower than the MIC for the majority of the distribution of *P. multocida* [34], it exceeds the MSC of 0.06 mg/L (Fig 3) and so may select for resistance. To target an exposure value below the MSC, a maximum cross-contamination limit should be 0.067 mg/kg or 0.67% of the target-dose batch.

The MSC value of 0.06 mg/L is 100-fold greater than the median florfenicol concentration found in animal wastewater/effluent across 32 sites in China [35] depicted in Fig 3. This suggests that enrichment of *floR*-carrying *P. multocida* (or *cfr-*carrying *E. Coli,* Fig 3B) is unlikely in these settings and are not significant reservoirs or transmission sites of florfenicol resistance. However, this study was carried out in neutral pH, with optimised conditions for bacterial growth. In acidic slurry *P. multocida* has been found to survive for 6 days if conditions are warm [36], and there is little degradation of florfenicol [37]. The MSC under these conditions could be different if there is further differential selective pressure on the susceptible vs resistant populations from the environment.

Another important consideration is the impact of polymicrobial communities on the MSC. Development of biofilms, and synergism can increase the ability of bacteria including some strains of *P. multocida* to survive under AMD selective pressure [27,38–40]. Klümper *et. al* [41] found a 43x greater gentamicin MSC for *E. coli* in the presence of a polymicrobial faecal community, which brought this in line with the MIC. Since *P. multocida* is most commonly part of polymicrobial infections and environments, the next step from determination of the MSC as a single species culture achieved here should be to assess the effect of interactions with other bacterial species.

Florfenicol resistance is commonly found on plasmids in *Pasteurella* and other species [7,42,43]. Chromosomal integration, as performed in previous MSC studies [25,27,28,41], allows the effect of the resistance gene on fitness cost to be separated from the plasmid. However, it is not necessary to represent resistance selection in the real-world. The *floR* carrying plasmid used here was obtained from a wildtype isolate and is highly similar (96% coverage of contig 4, 99.6% identity, S2 Fig) to previously characterised pCCK381 plasmid isolated from *P. multocida* [44]. The co-culture study here is representative of naturally occurring HGT of resistance to a previously susceptible isolate. The *floR* plasmid contains at least 1 mobilisation genes (S1 Fig); during the selection process it is possible that the resistant population is increased via HGT, however the contribution of this to the overall change in ratio of resistant to susceptible populations is expected to be low compared to the impact of suppression of the growth rate of the susceptible population from florfenicol. The MSC determined here cannot be exclusively attributed to the presence/expression of *floR*. Chromosomal introduction of *floR* would be required to assess the impact of the plasmid backbone. However, techniques for targeted genetic modification of *P. multocida* have not been routinely described. This research could be expanded upon to introduce fluorescence genes + /- *floR* into *P. multocida*, allowing the ratio of resistant to susceptible isolates to be determined by flow-cytometry as described for *E. coli* and *S. typhimurium* [25].

This study into sub-inhibitory resistance selection to florfenicol in *P. multocida* builds on the previous research into the MSC by defining an MSC in a novel pathogen and developing the methods to achieve this. These methods can be easily extrapolated across bacterial species, including fastidious organisms, to enable a greater application and understanding of the MSC across the microbiome. The MSC of 0.06 mg/L has important implications for in-feed contamination, however, it appears that resistance selection in the environment is unlikely to occur. Further research into polymicrobial communities and local environments would increase the understanding of how this research relates to resistance dissemination in the real world.

## Materials and methods

### Isolates

Florfenicol susceptible (PM6) and resistant (PM4) clinical isolates of *Pasteurella multocida* were obtained from post-mortem samples from pigs with respiratory disease in Spain between 2020 and 2022. Samples were recovered on MHF agar (cation-adjusted Mueller-Hinton agar (Oxoid, UK) + 5% defibrinated horse blood (TCS Bioscience, UK) + 20 mg/L β-nicotinamide adenine dinucleotide (NAD) (Glentham Life Science, UK)).

Whole genome sequencing (WGS) using a hybrid approach (combining Illumina short reads and Oxford Nanopore Technologies long-reads to enhance read depth, promote identification of plasmids, and identify location of resistance genes) and bioinformatics (assembly using Unicycler version 0.4.0, and contig annotation with Prokka version 1.11) was performed by MicrobesNG (UK). The assembled genome for florfenicol susceptible PM6 and florfenicol-resistant PM4 were assessed for AMR genes using the Resistance Gene Identifier from the Comprehensive Antibiotic Resistance Database [45] (Accessed 05/02/2024).

### Florfenicol

Stock florfenicol (Produlab Pharma, Netherlands) solutions were prepared at 4 mg/mL in 95% ethanol. Subsequent dilution was performed in MHF broth.

### Minimum inhibitory concentration

Florfenicol MICs were first determined by 2-fold microdilution according to CLSI guidelines [46] in Muller-Hinton Fastidious (MHF) broth. *Streptococcus pneumoniae* (ATCC49619) was used as a quality control. Precise MICs were then determined by performing the above procedure with florfenicol concentrations at 20% steps from 0.25–2 mg/L (i.e., **0.25**, 0.3, 0.35, 0.4, 0.45, **0.5** mg/L, 2-fold concentration in bold). MICs were determined as the lowest concentration with no visible pellet or turbidity compared to the negative control.

### Generation and characterisation of *floR* transformants (Fig 4)

#### Plasmid extraction.

Plasmids were extracted from PM4 using a Monarch Plasmid DNA Miniprep Kit (New England Biolabs, MA, USA) following manufacturer’s instructions, quantification using a Denovix DS-11 spectrophotometer (DeNovix, DE, USA), diluted to 30 ng/μL with ultrapure H_2_O and stored at −20°C.

**Fig 4:**
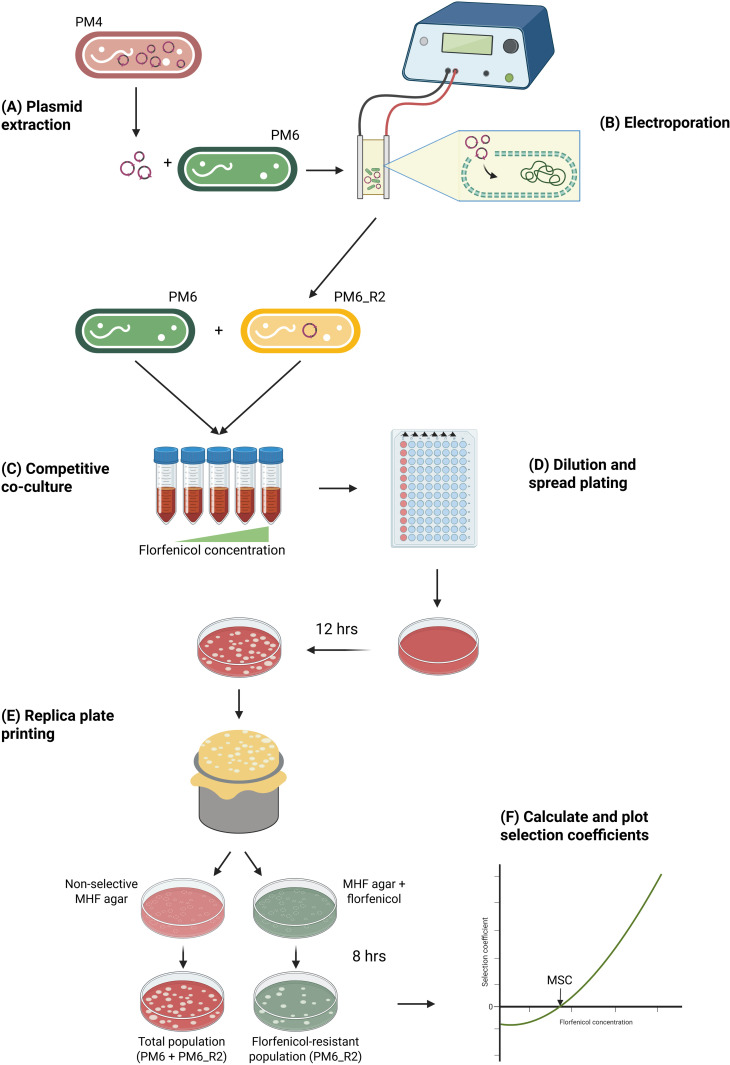
Generation of floR transformants and co-culture for MSC determination. Plasmids containing floR (and other AMR genes) were obtained from PM4 (A) and used to transform PM6 by electroporation **(B)**. Florfenicol resistant transformant PM6_R2 was co-cultured with (susceptible) PM6 at sub-MIC_SUSC_ florfenicol concentrations from 1/20 to 1/2 x MIC_SUSC_ (0.0175 to 0.175 mg/L) **(C)**. Every 24 hours 1000-fold dilution into fresh media at the same florfenicol concentration was performed and each culture was sampled in triplicate, diluted to 10^−6^ and spread on non-selective MHF agar **(D)**. After 12 hours incubation, replica plate printing was performed onto non-selective and selective MHF agar at florfenicol concentration 3 x MIC_SUSC_ **(E)**. Colonies were counted on each plate after 5- and 8-hours incubation. Selection coefficients (sc) were calculated for each time point (t) based on the ratio of resistant to susceptible bacteria according to sc = ln(Ratio_(t)_/Ratio_(0)_)/t. Interpolation of a model fitted to the selection coefficients as a function of florfenicol concentration determined the MSC where sc equals 0 (F).

#### Electroporation.

An electroporation protocol for *P. multocida* was adapted from Jablonski *et. al.* [47]. Electrocompetent PM6 cells were prepared from overnight cultures in 0.05% NaCl lysogeny broth + 0.2% glucose. Twenty mLs of culture were washed and resuspended in ice-cold glycerol to a concentration of approximately (1 x 10^10^ CFU/mL). Forty μL of the prepared cell suspension was electroporated (50 μF, 129 Ω, 12.5 kV/cm) with 5 ng plasmid DNA from PM4 using a BTX Electrocell Manipulator 600 (BTX, MA, USA).

Samples were recovered in 200 μL MHF at 37°C for 3 hours before selection on MHF agar supplemented 3 x MIC_SUSC_ (1.05 mg/L) florfenicol (Fig 4).

#### Polymerase chain reaction.

Polymerase chain reaction (PCR) was performed to identify presence of *floR* and *sul2* genes. Bacterial DNA templates were prepared by thermal lysis at 95°C for 10 minutes.

Amplification reactions (26 μL) contained: 13 μL DreamTaq Green PCR master mix, 10.5 μL ultrapure water, 0.5 μL each forward and reverse primer (Table 1) and 2 μL DNA template [48,49]. The cycling program comprised initial denaturation at 94°C for 5 minutes, 30x amplification cycle of: denaturation at 94°C for 40 s, annealing at 55°C for 45 seconds, and elongation at 72°C for 50 seconds, with final elongation at 72°C for 10 minutes. An Applied Biosystems™ SimpliAmp™ Thermal cycler (Fisher scientific, Loughborough, UK) was used for all reactions. PCR products were detected by gel electrophoresis (85V for 45 minutes) in agarose 1.5% w/v gels with SYBR™ Safe DNA gel stain. The molecular size marker was 100 bp plus GeneRuler DNA Ladder mix. All reagents were obtained from ThermoFisher Scientific (MA, USA).

**Table 1:** PCR primers for identification of floR and sul2.

Target	Primer sequence (5′-3′)	Amplicon (bp)	Reference
*floR*	F-ACGTTTATGCCAACCGTCCT R- CATTACAAGCGCGACAGTGG	398	Li *et al.* [48]
*sul2*	F- GCAGGCGCGTAAGCTGA R- GGCTCGTGTGTGCGGATG	657	Jiang *et al.* [49]

#### Phenotypic AMR screening.

Transformants were screened for non-florfenicol AMR phenotypes present in plasmid donor strain PM4 by disc diffusion of tetracycline (30 μg, Oxoid), sulphamethoxazole/trimethoprim (25 μg, Oxoid), erythromycin (15 μg, MAST), and streptomycin (10 μg, Oxoid) according to CLSI guidelines [46]. Classification of susceptible versus resistant was based on published ECOFF from EUCAST ([50] accessed 24/06/2024, available for tetracycline and sulphamethoxazole/trimethoprim) and comparison to control strains PM6 and PM4.

### Competition experiments

To determine the MSC for *floR*-mediated resistance, competitive growth assays for PM6 vs PM6_R2 at sub-MIC florfenicol concentrations were performed in triplicate.

Independent 15 mL cultures were prepared in MHF with florfenicol concentrations of 0, 0.0175 (1/20 x MIC), 0.0525 (1/6.6 x or 3/20 x MIC), 0.0875 (1/4 x MIC), and 0.175 (1/2 x MIC) mg/L with an initial inoculum of 2.5 x 10^5^ CFU/mL of each PM6 and PM6_R2. Each culture was serially passaged by 1000-fold dilution every 24 hours. At time 0, 24 and 48 hours, 3 samples from each condition prior to passage were diluted by serial 10-fold dilution in PBS. Eighty µL from the 100-fold dilution at time 0 and 1x10^6^-fold dilution at subsequent time-points were spread onto MHF agar and incubated at 37°C for 12 hours. Colonies were transferred to fresh non-selective MHF agar and florfenicol-selective MHF agar (3x MIC_SUSC_, 1.05 mg/L) by replica plate printing and incubated for 8 hours. CFU on each plate were counted after 5 and 8 hours of incubation to determine total and resistant populations. Susceptible population was taken as the total count minus the resistant count.

The ratio of resistant to susceptible colonies (*Ratio*) was used to calculate the selection coefficient (*sc*) for each concentration at each time point (*t*) using the regression model:


$$\begin{array}{c} sc=\frac{\ln(\frac{{Ratio}_{\left(t\right)}}{{Ratio}_{\left(0\right)}})}{t} \end{array}$$

where if the proportion of each population does not change: Ratio_(t)_ = Ratio_(0),_ then *sc *= *ln*(1) = 0. If the ratio at time *t* favours the resistant population, then the natural log takes a positive value, as does *sc*.

The calculated selection coefficients were plotted as a function of florfenicol concentration using GraphPad Prism v10.0.0 for Windows [51]. The MSC was determined by interpolation using a nonlinear regression model to define the concentration at which the selection coefficient equals 0.

### Spontaneous mutation in *P. multocida*

#### Assessment for hypermutator phenotype: rifampicin assay.

**Fig 5:**
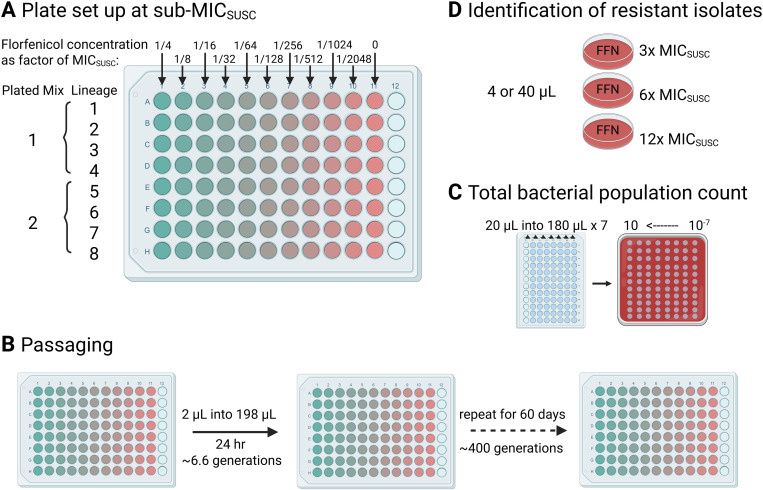
Serial passaging. Eight lineages of PM6 were passaged at 10 different concentrations of florfenicol below MIC_SUSC_ with a growth control **(A)**. Each lineage was passaged by 100-fold dilution into fresh media of the same concentration every 24 hours for 60 days **(B)**. Lineages 1−4 and 5−8 were combined for each concentration and these 22 mixtures (2 mixtures from 11 conditions) were screened for resistant isolates (D) and average total bacterial population enumerated (C) every 24 hours (approx. 6.6 generations) for the first 10 days and subsequently every 5 days (approx. 30 generations). Screening was performed by plating 4 μL (Mix 1, approximately 2 x 10^6^ CFU) or 40 μL (Mix 2, approx. 2 x 10^7^ CFU) onto MHF agar containing florfenicol at 3x, 6x, and 12x MIC_SUSC_ and incubating for 48 hours at 37°C. Plates were assessed for resistant isolate growth at 24 and 48 hours. Bacterial population enumeration was performed by dilution to 10^−7^ in PBS and spot plating onto non-selective MHF agar with back calculation from CFU count after overnight incubation.

The spontaneous mutation rate of PM6 was determined by fluctuation rate assay with rifampicin (Glentham Life Sciences, UK). With this method, the distribution of mutant numbers in parallel cultures allows the estimation of the number of mutations per culture and the differential growth rate between mutant and wild-type cells. Analysis of these parameters according to the Luria-Delbrück distribution allows the computation of the mutation rate [52]. The assay was carried out as previously described [53], with 24 independent replicates of PM6 with an initial inoculum of approximately 2000 CFU in 250 μL. After overnight growth at 37°C, 100 μL from each independent culture was plated on MHF-agar containing 100 mg/L of rifampicin. Serial dilution and plating for total bacterial count, as described in Fig 5C, was also performed. After overnight incubation, the number of CFU was counted. The web-tool “bz-rates” [54] was used to compute the mutation rate according to the Luria-Delbrück distribution by inputting the number of mutants per total colonies plated and the fraction of culture plated on selective agar (0.4).

#### Serial passaging to determine the emergence of resistant mutants.

To assess for the *de novo* evolution of resistance from a susceptible strain at sub-MIC florfenicol concentrations, a protocol was adapted from Pereira *et al.* [5]. Eight lineages of PM6 were serially passaged every 24 hours, as shown in Fig 5, for 400 generations.

A 2-fold florfenicol dilution series ranging from 1/4 x MIC_SUSC_ (0.0875 mg/L) to 1/2048 x MIC_SUSC_ (1.7 x 10^-4^ mg/L) and a no-drug control was prepared in 96-well plates (Fig 5). Each well of the first plate was inoculated by 100-fold dilution from an overnight culture (37°C, shaking) of PM6 (target inoculum size of 5 x 10^7^ CFU/mL), and incubated at 37°C for 24 hours. Each serial passage corresponds to approximately 6.7 generations of growth Subsequently, every 24 hours, each well of the incubated plate was mixed thoroughly by pipetting 50 µL up-and-down, before transferring 2 µL (100-fold dilution) to a fresh pre-warmed plate and incubated at 37°C for 24 hours (Fig 5B). Enumeration (Fig 5C) and presence of resistant isolates (Fig 5D) was performed as a population-level assessment of four lineages of the same selective condition combined, (i.e., 2 samples combining four lineages, as in Fig 5A), per florfenicol concentration per plate.

#### Luria-Delbrück assay.

To assess if spontaneous florfenicol resistance mutation was possible, a Luria-Delbrück assay [55] was conducted with PM6. Ten independent 20 mL MHF cultures of PM6 were incubated for 24 hours with a starting inoculum of 8000 CFU/mL. Bacterial suspensions were centrifuged at 5000 x g for 5 minutes and supernatant discarded. Each bacterial pellet was resuspended in 400 μL MHF and spread equally onto florfenicol selective MHF-agar at 2x and 4x the MIC of PM6 (0.7 and 1.4 mg/L respectively). Agar plates were incubated at 37°C for 48 hours and assessed for resistant isolate growth at 24 and 48 hours.

## Supporting information

S1 Fig Map of contig 4 from PM4. This contig contains the *floR* gene, and genes encoding mobilisation protein mobA_2, and replication protein repA as annotated during WGS bioinformatics at MicrobesNG. “Hypothetical proteins” were searched by basic local alignment at NCBI and proteins identified are given in brackets. These include secondary replication protein repC and mobilisation protein mobC.
(TIF)

S2 Fig Alignment of plasmid pCCK381 (top sequence) and pPM4 (bottom “query sequence”). The regions encoding *mobA*, *repA*, *repC*, and *floR* in plasmid pCCK381 (top sequence) are also present in pPM4 (bottom sequence, the plasmid obtained from PM4 and electroporated into PM6 to induce florfenicol resistance as PM6_R2).
(TIF)

S3 Fig OD600 growth curves demonstrate little difference in growth rate between susceptible and resistant isolates measured by optical density. Mean and standard deviation of growth curves as measured by OD600 for 75 independent cultures of PM6 (susceptible wild-type isolate), and PM6_R2 (florfenicol resistant transformed mutant) in CAMHB. Time 0 is normalised for each replicate to when OD600 > 0.1 (background OD600 of CAMHB).
(TIF)

## References

[1] Toutain P-L, Ferran AA, Bousquet-Melou A, Pelligand L, Lees P. Veterinary medicine needs new green antimicrobial drugs. Front Microbiol. 2016;7:1196. doi: 10.3389/fmicb.2016.01196 27536285 PMC4971058

[2] Winter M, Buckling A, Harms K, Johnsen PJ, Vos M. Antimicrobial resistance acquisition via natural transformation: context is everything. Curr Opin Microbiol. 2021;64:133–8. doi: 10.1016/j.mib.2021.09.009 34710742

[3] Global Leaders Group on Antimicrobial Resistance. Priorities of the Global Leaders Group on AMR. 2023.

[4] UN Environment Programme. Bracing for Superbugs: Strengthening environmental action in the One Health response to antimicrobial resistance. 2023. Available from: https://www.unep.org/resources/superbugs/environmental-action#:~:text=The%20report%20Bracing%20for%20Superbugs,key%20part%20of%20the%20solution.

[5] Pereira C, Warsi OM, Andersson DI. Pervasive selection for clinically relevant resistance and media adaptive mutations at very low antibiotic concentrations. Mol Biol Evol. 2023;40(1):msad010. doi: 10.1093/molbev/msad010 36627817 PMC9887637

[6] Piorunek M, Brajer-Luftmann B, Walkowiak J. Pasteurella multocida infection in humans. Pathogens. 2023;12(10):1210. doi: 10.3390/pathogens12101210 37887726 PMC10610061

[7] Michael GB, Kadlec K, Sweeney MT, Brzuszkiewicz E, Liesegang H, Daniel R, et al. ICEPmu1, an integrative conjugative element (ICE) of Pasteurella multocida: structure and transfer. J Antimicrob Chemother. 2012;67(1):91–100. doi: 10.1093/jac/dkr411 22001176

[8] Michael GB, Bossé JT, Schwarz S. Antimicrobial resistance in pasteurellaceae of veterinary origin. In: Antimicrobial Resistance in Bacteria from Livestock and Companion Animals. 2018. p. 331–63. 10.1128/microbiolspec.arba-0022-2017 PMC11633590 29916344

[9] Cannon M, Harford S, Davies J. A comparative study on the inhibitory actions of chloramphenicol, thiamphenicol and some fluorinated derivatives. J Antimicrob Chemother. 1990;26(3):307–17. doi: 10.1093/jac/26.3.307 2228823

[10] Yao X, Song Q, Zhu W, Wei J, Shao D, Liu K, et al. Characterization of small plasmids carrying florfenicol resistance gene floR in Actinobacillus pleuropneumoniae and Pasteurella multocida isolates from swine in China. Front Vet Sci. 2023;10:1084491. doi: 10.3389/fvets.2023.1084491 36793377 PMC9922843

[11] Kehrenberg C, Wallmann J, Schwarz S. Molecular analysis of florfenicol-resistant Pasteurella multocida isolates in Germany. J Antimicrob Chemother. 2008;62(5):951–5. doi: 10.1093/jac/dkn359 18786941

[12] WHO. Critically important antimicrobials for human medicine. 6th ed. Geneva: World Health Organization; 2019.

[13] EFSA Panel on Biological Hazards (BIOHAZ), Koutsoumanis K, Allende A, Alvarez-Ordóñez A, Bolton D, Bover-Cid S, et al. Maximum levels of cross-contamination for 24 antimicrobial active substances in non-target feed. Part 7: Amphenicols: florfenicol and thiamphenicol. EFSA J. 2021;19(10):e06859. doi: 10.2903/j.efsa.2021.6859 34729087 PMC8546524

[14] EFSA Panel on Biological Hazards (BIOHAZ), Koutsoumanis K, Allende A, Alvarez-Ordóñez A, Bolton D, Bover-Cid S, et al. Maximum levels of cross-contamination for 24 antimicrobial active substances in non-target feed. Part 1: Methodology, general data gaps and uncertainties. EFSA J. 2021;19(10):e06852. doi: 10.2903/j.efsa.2021.6852 34729081 PMC8547316

[15] Zhang J, Hu K, Chen Z, Deng H, Huang X. Effect of plasmid pSD11 on the fitness of Escherichia coli in sub-minimum inhibitory concentration of florfenicol. Chinese J Appl Environ Biol. 2019;25:1211–4. doi: 10.19675/j.cnki.1006-687x.2018.12045

[16] Hibbing ME, Fuqua C, Parsek MR, Peterson SB. Bacterial competition: surviving and thriving in the microbial jungle. Nat Rev Microbiol. 2010;8(1):15–25. doi: 10.1038/nrmicro2259 19946288 PMC2879262

[17] Andersson DI, Hughes D. Microbiological effects of sublethal levels of antibiotics. Nat Rev Microbiol. 2014;12(7):465–78. doi: 10.1038/nrmicro3270 24861036

[18] Wistrand-Yuen E, Knopp M, Hjort K, Koskiniemi S, Berg OG, Andersson DI. Evolution of high-level resistance during low-level antibiotic exposure. Nat Commun. 2018;9(1):1599. doi: 10.1038/s41467-018-04059-1 29686259 PMC5913237

[19] Limited NL. Summary of product characteristics Alamycin LA 200 mg/ml solution for injection for cattle, sheep and pigs. United Kingdom; 2022.

[20] SL GVH. Summary of product characteristics amoxicillin global vet health 500 mg/g, powder for use in drinking water For chickens, turkeys, ducks and pigs. Spain; 2020.

[21] Veterinary Medicines Directorate AaPHA. UK Veterinary Antimicrobial Resistance and Sales Surveillance 2023. United Kingdom; 2024.

[22] Mulchandani R, Wang Y, Gilbert M, Van Boeckel TP. Global trends in antimicrobial use in food-producing animals: 2020 to 2030. PLOS Glob Public Health. 2023;3(2):e0001305. doi: 10.1371/journal.pgph.0001305 36963007 PMC10021213

[23] Klein EY, Impalli I, Poleon S, Denoel P, Cipriano M, Van Boeckel TP, et al. Global trends in antibiotic consumption during 2016-2023 and future projections through 2030. Proc Natl Acad Sci U S A. 2024;121(49):e2411919121. doi: 10.1073/pnas.2411919121 39556760 PMC11626136

[24] Joint Formulary Commmittee. Chloramphenicol. In: British National Formulary (online) [Internet]. London: BMJ and Pharmaceutical Press; 2024 [cited 16/07/2024]. Available from: https://bnf.nice.org.uk/drugs/chloramphenicol/

[25] Gullberg E, Cao S, Berg OG, Ilbäck C, Sandegren L, Hughes D, et al. Selection of resistant bacteria at very low antibiotic concentrations. PLoS Pathog. 2011;7(7):e1002158. doi: 10.1371/journal.ppat.1002158 21811410 PMC3141051

[26] Imazaki PH, Voisin B, Arpaillange N, Roques BB, Dordet-Frisoni E, Dupouy V, et al. The sub-MIC selective window decreases along the digestive tract: determination of the minimal selective concentration of oxytetracycline in sterilised intestinal contents. Front Microbiol. 2024;15:1377159. doi: 10.3389/fmicb.2024.1377159 38946898 PMC11211281

[27] Hjort K, Fermér E, Tang P-C, Andersson DI. Antibiotic minimal selective concentrations and fitness costs during biofilm and planktonic growth. mBio. 2022;13(3):e0144722. doi: 10.1128/mbio.01447-22 35695458 PMC9239065

[28] Gullberg E, Albrecht LM, Karlsson C, Sandegren L, Andersson DI. Selection of a multidrug resistance plasmid by sublethal levels of antibiotics and heavy metals. mBio. 2014;5(5):e01918-14. doi: 10.1128/mBio.01918-14 25293762 PMC4196238

[29] Beal J, Farny NG, Haddock-Angelli T, Selvarajah V, Baldwin GS, Buckley-Taylor R, et al. Robust estimation of bacterial cell count from optical density. Commun Biol. 2020;3(1):512. doi: 10.1038/s42003-020-01127-5 32943734 PMC7499192

[30] European Commission D-GfHaFS. Commission Delegated Regulation (EU) 2024/1229 of 20 February 2024 supplementing Regulation (EU) 2019/4 of the European Parliament and of the Council by establishing specific maximum levels of cross-contamination of antimicrobial active substances in non-target feed and methods of analysis for these substances in feed. In: SANTE, editor. 2024.

[31] MSD Animal Health. Summary of Product Characteristics Nuflor 40 mg/g Premix for Medicated Feeding Stuff for Swine Buckinghmashire, UK. 2020. Available from: https://www.vmd.defra.gov.uk/productinformationdatabase/product/A006949

[32] Liu J, Fung K-F, Chen Z, Zeng Z, Zhang J. Pharmacokinetics of florfenicol in healthy pigs and in pigs experimentally infected with Actinobacillus pleuropneumoniae. Antimicrob Agents Chemother. 2003;47(2):820–3. doi: 10.1128/AAC.47.2.820-823.2003 12543702 PMC151723

[33] Lei Z, Liu Q, Yang S, Yang B, Khaliq H, Li K, et al. PK-PD Integration Modeling and Cutoff Value of Florfenicol against Streptococcus suis in Pigs. Front Pharmacol. 2018;9:2. doi: 10.3389/fphar.2018.00002 29387013 PMC5776115

[34] EUCAST. Data from the EUCAST MIC distribution website: European Committee on Antimicrobial Susceptibility Testing; [cited 2024 20/07/2024]. Available from: https://mic.eucast.org/

[35] Wei R, Ge F, Chen M, Wang R. Occurrence of ciprofloxacin, enrofloxacin, and florfenicol in animal wastewater and water resources. J Environ Qual. 2012;41(5):1481–6. doi: 10.2134/jeq2012.0014 23099939

[36] Thomson CM, Chanter N, Wathes CM. Survival of toxigenic Pasteurella multocida in aerosols and aqueous liquids. Appl Environ Microbiol. 1992;58(3):932–6. doi: 10.1128/aem.58.3.932-936.1992 1575496 PMC195358

[37] Nightingale J, Carter L, Sinclair CJ, Rooney P, Dickinson M, Tarbin J, et al. Assessing the influence of pig slurry pH on the degradation of selected antibiotic compounds. Chemosphere. 2022;290:133191. doi: 10.1016/j.chemosphere.2021.133191 34896423

[38] Sun Y, Mao W, Cao J, Guo HP, Jianguo S, Yin K, et al. Chinese medicine monomers inhibit biofilm formation in multidrug-resistant P. multocida isolated from cattle respiratory infections. Pakistan Veter J. 2024;44(4).

[39] Prajapati A, Chanda MM, Dhayalan A, Yogisharadhya R, Chaudhary JK, Mohanty NN, et al. Variability in in vitro biofilm production and antimicrobial sensitivity pattern among Pasteurella multocida strains. Biofouling. 2020;36(8):938–50. doi: 10.1080/08927014.2020.1833192 33059484

[40] Little W, Black C, Smith AC. Clinical implications of polymicrobial synergism effects on antimicrobial susceptibility. Pathogens. 2021;10(2):144. doi: 10.3390/pathogens10020144 33535562 PMC7912749

[41] Klümper U, Recker M, Zhang L, Yin X, Zhang T, Buckling A, et al. Selection for antimicrobial resistance is reduced when embedded in a natural microbial community. ISME J. 2019;13(12):2927–37. doi: 10.1038/s41396-019-0483-z 31384011 PMC6864104

[42] Peng Z, Wang X, Zhou R, Chen H, Wilson BA, Wu B. Pasteurella multocida: genotypes and genomics. Microbiol Mol Biol Rev. 2019;83(4):e00014-19. doi: 10.1128/MMBR.00014-19 31484691 PMC6759666

[43] Schwarz S, Kehrenberg C, Doublet B, Cloeckaert A. Molecular basis of bacterial resistance to chloramphenicol and florfenicol. FEMS Microbiol Rev. 2004;28(5):519–42. doi: 10.1016/j.femsre.2004.04.001 15539072

[44] Kehrenberg C, Schwarz S. Plasmid-borne florfenicol resistance in Pasteurella multocida. J Antimicrob Chemother. 2005;55(5):773–5. doi: 10.1093/jac/dki102 15814600

[45] Alcock BP, Huynh W, Chalil R, Smith KW, Raphenya AR, Wlodarski MA, et al. CARD 2023: expanded curation, support for machine learning, and resistome prediction at the Comprehensive Antibiotic Resistance Database. Nucleic Acids Res. 2023;51(D1):D690–d9. doi: 10.1093/nar/gkac920 36263822 PMC9825576

[46] CLSI. VET01-A4 Performance Standards for Antimicrobial Disk and Dilution Susceptibility Tests for Bacteria Isolated From Animals; Approved Standard—Fourth Edition. Wayne, PA: Clinical Laboratory Standards Institute; 2013.

[47] Jablonski L, Sriranganathan N, Boyle SM, Carter GR. Conditions for transformation of Pasteurella multocida by electroporation. Microb Pathog. 1992;12(1):63–8. doi: 10.1016/0882-4010(92)90066-w 1560754

[48] Li P, Zhu T, Zhou D, Lu W, Liu H, Sun Z, et al. Analysis of resistance to florfenicol and the related mechanism of dissemination in different animal-derived bacteria. Front Cell Infect Microbiol. 2020;10:369. doi: 10.3389/fcimb.2020.00369 32903722 PMC7438884

[49] Jiang H, Cheng H, Liang Y, Yu S, Yu T, Fang J, et al. Diverse mobile genetic elements and conjugal transferability of sulfonamide resistance genes (sul1, sul2, and sul3) in Escherichia coli isolates from Penaeus vannamei and pork from large markets in Zhejiang, China. Front Microbiol. 2019;10:1787. doi: 10.3389/fmicb.2019.01787 31428076 PMC6690019

[50] EUCAST. Data from the EUCAST MIC distribution website: European Committee on Antimicrobial Susceptibility Testing; [cited 24 June 2024]. Available from: https://mic.eucast.org/search/show-registration/42173?back=https://mic.eucast.org/search/?search%255Bmethod%255D%3Dmic%26search%255Bantibiotic%255D%3D-1%26search%255Bspecies%255D%3D280%26search%255Bdisk_content%255D%3D-1%26search%255Blimit%255D%3D50

[51] GraphPad Software. GraphPad Prism version 10.0.0 for Windows. Boston, Massachusetts, USA: GraphPad Software.

[52] Rosche WA, Foster PL. Determining mutation rates in bacterial populations. Methods. 2000;20(1):4–17. 10610800 10.1006/meth.1999.0901 PMC2932672

[53] Krašovec R, Richards H, Gomez G, Gifford DR, Mazoyer A, Knight CG. Measuring microbial mutation rates with the fluctuation assay. J Vis Exp. 2019;(153):10.3791/60406. doi: 10.3791/60406 31840662

[54] Gillet-Markowska A, Louvel G, Fischer G. bz-rates: A web tool to estimate mutation rates from fluctuation analysis. G3 (Bethesda). 2015;5(11):2323–7. doi: 10.1534/g3.115.019836 26338660 PMC4632052

[55] Blondeau JM, Fitch SD. Mutant prevention and minimum inhibitory concentration drug values for enrofloxacin, ceftiofur, florfenicol, tilmicosin and tulathromycin tested against swine pathogens Actinobacillus pleuropneumoniae, Pasteurella multocida and Streptococcus suis. PLoS One. 2019;14(1):e0210154. doi: 10.1371/journal.pone.0210154 30629633 PMC6328246

