# Supplementary figures and images for: Selection for florfenicol resistance at sub-MIC concentrations in Pasteurella multocida
Source: PLoS One. 2025 Jun 24;20(6):e0327115. doi: 10.1371/journal.pone.0327115 (PMC12186936; doi:10.1371/journal.pone.0327115)

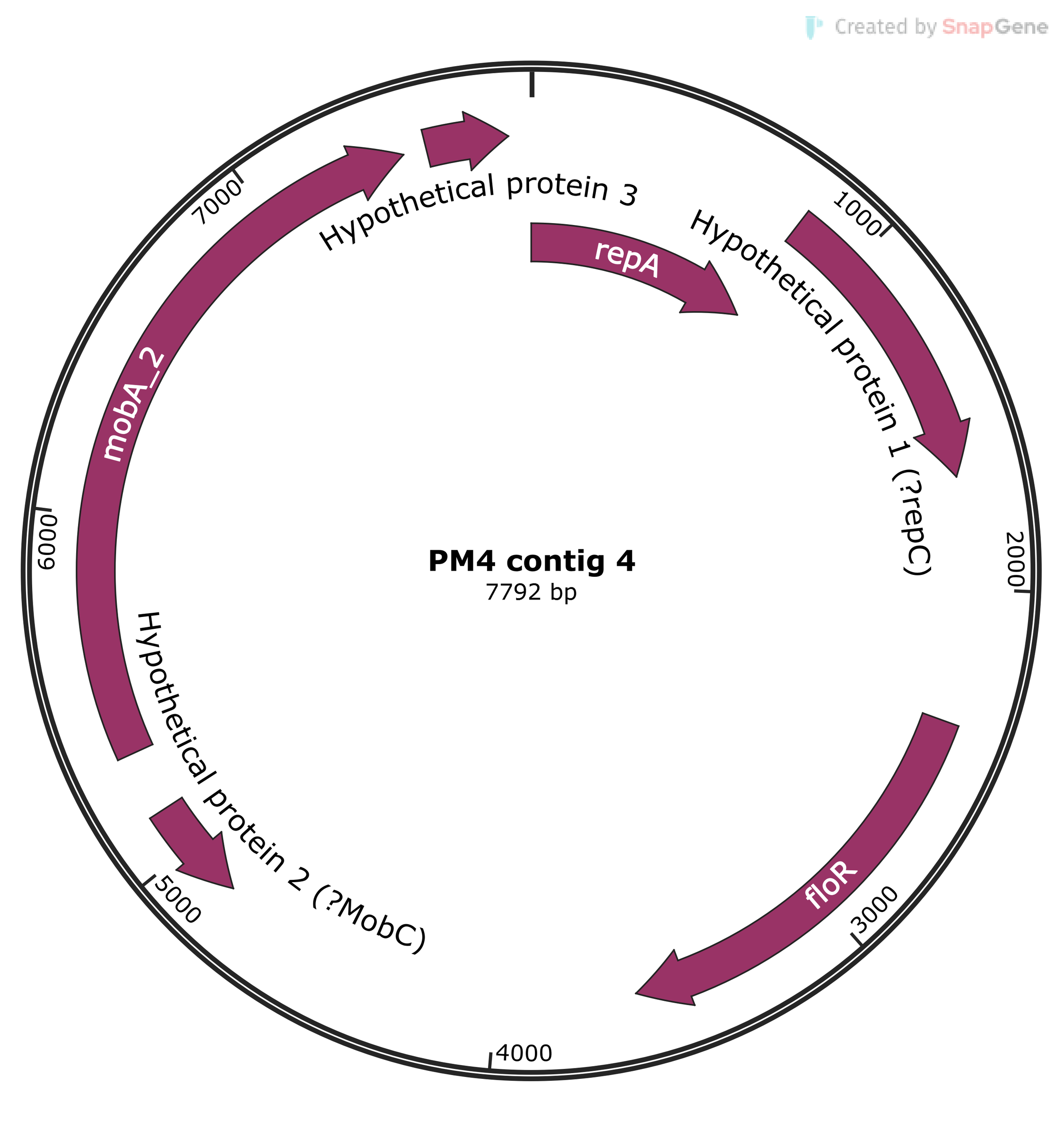

Supplement: S1 Fig — This contig contains the floR gene, and genes encoding mobilisation protein mobA_2, and replication protein repA as annotated during WGS bioinformatics at MicrobesNG. “Hypothetical proteins” were searched by basic local alignment at NCBI and proteins identified are given in brackets. These include secondary replication protein repC and mobilisation protein mobC. (TIF) [file pone.0327115.s001.tif]

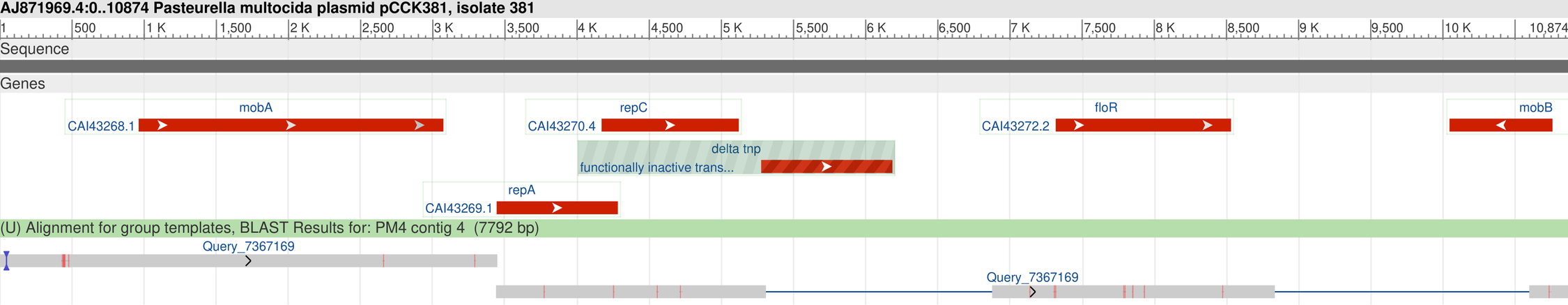

Supplement: S2 Fig — The regions encoding mobA, repA, repC, and floR in plasmid pCCK381 (top sequence) are also present in pPM4 (bottom sequence, the plasmid obtained from PM4 and electroporated into PM6 to induce florfenicol resistance as PM6_R2). (TIF) [file pone.0327115.s002.tif]

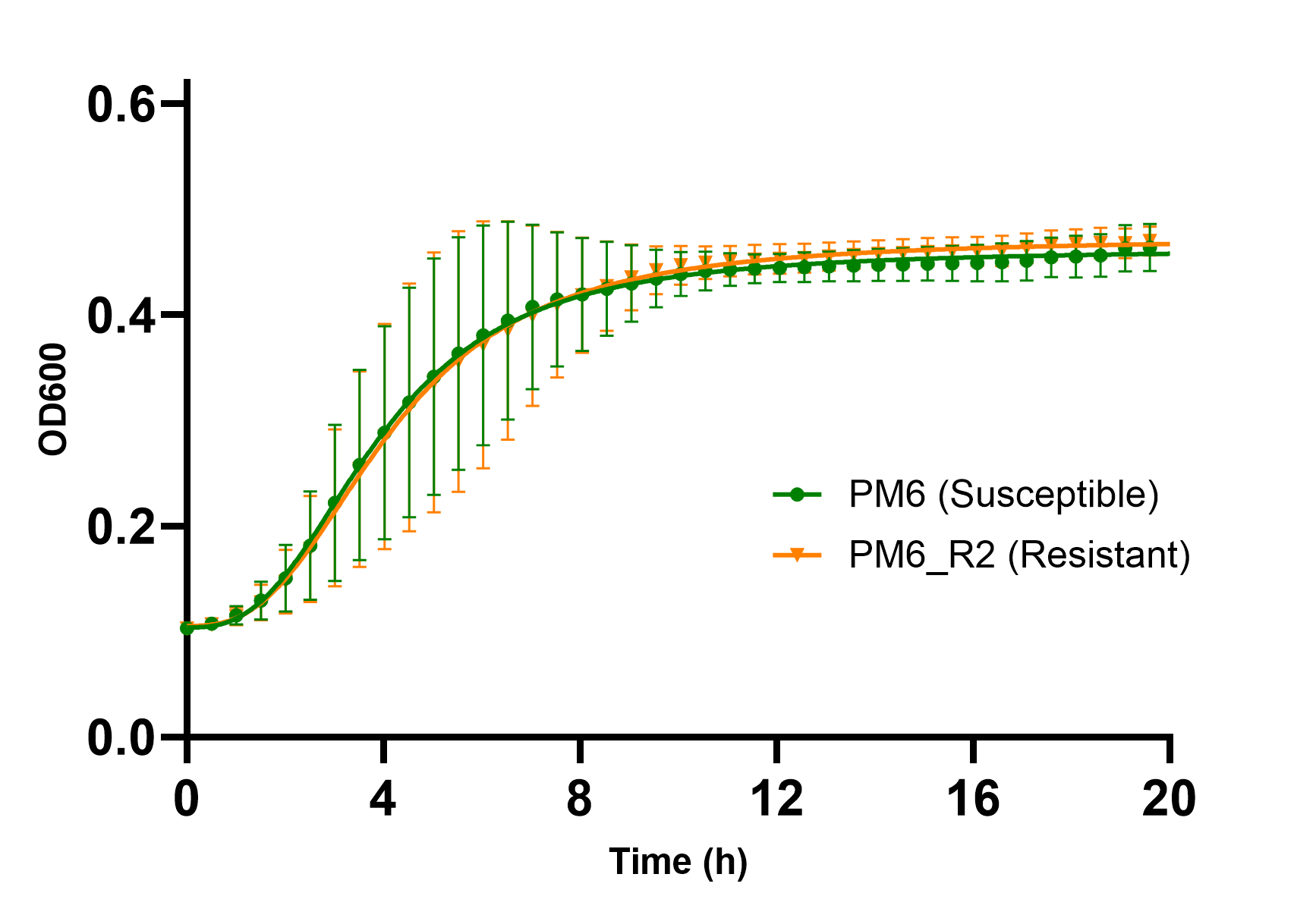

Supplement: S3 Fig — Mean and standard deviation of growth curves as measured by OD600 for 75 independent cultures of PM6 (susceptible wild-type isolate), and PM6_R2 (florfenicol resistant transformed mutant) in CAMHB. Time 0 is normalised for each replicate to when OD600 > 0.1 (background OD600 of CAMHB). (TIF) [file pone.0327115.s003.tif]
